# A Disturbance in the Force: Cellular Stress Sensing by the Mitochondrial Network

**DOI:** 10.3390/antiox7100126

**Published:** 2018-09-22

**Authors:** Robert Gilkerson

**Affiliations:** 1 Departments of Biology, The University of Texas Rio Grande Valley, Edinburg, TX 78539, USA; robert.gilkerson@utrgv.edu; 2 Clinical Laboratory Sciences, The University of Texas Rio Grande Valley, Edinburg, TX 78539, USA

**Keywords:** mitochondria, fusion, fission, OMA1, DRP, OPA1, stress, inflammation, cytokines, oxidative, bioenergetics

## Abstract

As a highly dynamic organellar network, mitochondria are maintained as an organellar network by delicately balancing fission and fusion pathways. This homeostatic balance of organellar dynamics is increasingly revealed to play an integral role in sensing cellular stress stimuli. Mitochondrial fission/fusion balance is highly sensitive to perturbations such as loss of bioenergetic function, oxidative stress, and other stimuli, with mechanistic contribution to subsequent cell-wide cascades including inflammation, autophagy, and apoptosis. The overlapping activity with *m*-AAA protease 1 (OMA1) metallopeptidase, a stress-sensitive modulator of mitochondrial fusion, and dynamin-related protein 1 (DRP1), a regulator of mitochondrial fission, are key factors that shape mitochondrial dynamics in response to various stimuli. As such, OMA1 and DRP1 are critical factors that mediate mitochondrial roles in cellular stress-response signaling. Here, we explore the current understanding and emerging questions in the role of mitochondrial dynamics in sensing cellular stress as a dynamic, responsive organellar network.

## 1. Mitochondria Balance Structural Dynamics with Bioenergetic Function

**Figure 1:**
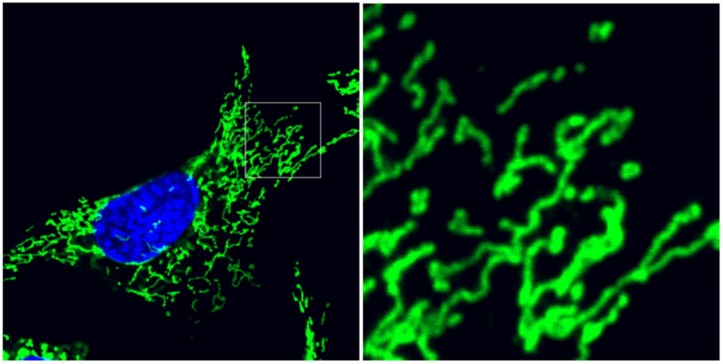
Mitochondrial ultrastructure balances between interconnection and division. Mouse embryonic fibroblasts immunolabeled for mitochondrial translocase of the outer membrane 20 (TOM20) outer membrane protein (green) and counterstained with diaminophenylindole (DAPI) (blue) for nuclei. Detail image depicts the pleiomorphic balance of isolated, spherical organelles and interconnected reticular mitochondria. Cellular stress induces remodeling of mitochondrial fusion and fission dynamics, controlled by overlapping activity with *m*-AAA protease 1 (OMA1) and dynamin-related protein 1 (DRP1), respectively.

Within eukaryotic cells, mitochondria occupy an essential role as an organellar network integrally responsible contributing to cellular homeostasis. Rather than an inert collection of vesicular bioenergetic ‘batteries’, this organellar network dynamically regulates its structural organization within the cell, balancing between two ultrastructural states: mitochondria may be maintained as an elaborately interconnected reticulum, or as a collection of independent organelles, (Figure 1). While these sensitive organellar dynamics are a fascinating biology in and of themselves, emerging research suggests that mitochondrial dynamics are inextricably linked to bioenergetics and adenosine triphosphate (ATP) production, as well as cell-wide signaling networks. In fact, the sensitive nature of mitochondrial dynamics places them as a key early indicator of cellular stress.

First described as “thread-like granules” in Greek, Benda first coined the term mitochondria in 1898. The advent of transmission electron microscopy (TEM) provided the first visualization of the mitochondrial outer and inner membranes, as well as the matrix and intermembrane space compartments by Palade [1] and Sjostrand [2]. The striking clarity of thin-section electron microscopy delineated the double membrane structure as we understand it today, leading to the canonical textbook image of mitochondria. The overall ultrastructure of mitochondria, however, remained elusive. Skulachev and co-workers rigorously explored the reticular nature of mitochondrial morphology through three-dimensional reconstruction of TEM images [3]. Advances in confocal fluorescence microscopy and electron tomography provided new perspectives on mitochondrial organization, revealing mitochondria to exist as a reticular organellar network arrayed throughout the cell [4,5,6]. Improved time-lapse imaging using mitochondrially-targeted green fluorescent protein demonstrated that mitochondria can both fuse and divide, with profound implications for the inheritance of the mitochondrial genome [7]. Mitochondrial DNA, organized into packaging units called nucleoids, are arranged at regular intervals throughout the mitochondrial network [8,9], ensuring that mitochondrial DNA (mtDNA)-derived content is available throughout the network and allowing for distribution of mtDNA during cell division. These dynamics involve cytoskeletal interactions with the cytoskeleton [10]. This homeostatic balance of interconnection and division is inseparably connected with mitochondrial bioenergetics in an elegant organellar structure/function relationship.

## 2. Mitochondrial Dynamics are Hand-in-hand with Bioenergetic Function

To carry out ATP production, proteins encoded on both nuclear and mtDNA are coordinately assembled into the five complexes of oxidative phosphorylation (OxPhos) at the inner membrane. While the vast majority of mitochondrial proteins are encoded by nuclear genes and subsequently imported to the mitochondria, mtDNA encodes only 12 polypeptides. Despite this, the mtDNA-encoded subunits are required for assembly and function of the OxPhos complexes and effective mitochondrial bioenergetics. Complexes I, II, III, and IV sequentially transfer electrons from nicotinamide adenine dinucleotide (NADH) and Flavin adenine dinucleotide (FADH_2_) to pump protons out of the matrix, thus creating the electrical and chemical gradient of the mitochondrial transmembrane potential (Δψ_m_). Complex V, the F_1_F_0_ ATP synthase, allows protons to diffuse down the gradient to efficiently coalesce ADP and Pi to ATP [11]. This bioenergetic function is required for effective fission/fusion balance. Genetic and pharmacological models of mitochondrial dysfunction both show an inability to maintain an interconnected mitochondrial reticulum: cells lacking mtDNA are deficient in mitochondrial ATP production, while protonophores such as carbonyl cyanide m-chlorophenyl hydrazone (CCCP) dissipate Δψ_m_. Both cause mitochondrial fragmentation, with a marked inability to maintain mitochondrial interconnection [12,13]. These findings are demonstrated the hand-in-hand nature of mitochondrial fission/fusion structural dynamics with bioenergetic function, with mtDNA arrayed throughout the network. In cells carrying heteroplasmic populations of mtDNA variants, the mitochondrial network frequently comprises a mixture of functional and non-functional organelles, in which organelles carrying different mtDNAs may transcomplement each other through fusion [14], while fission may isolate nonfunctional organelles for autophagic degradation [15]. MtDNA nucleoids tightly maintain their genetic composition, rather than exchanging mtDNAs [16]; fission/fusion dynamics allow the rapid segregation of mtDNA genotypes that can occur in heteroplasmic cell settings [17,18]. These findings collectively demonstrated that mitochondria are not a static set of energetic organelles, but delicately balance structural dynamics and metabolic function. Further, this dynamic network also emerged as a critical mediator of cellular stress response, with integral mechanistic roles in the life and death of the cell.

## 3. Mitochondria in Cellular Homeostasis

Mitochondrial biology was revolutionized in the mid-1990s when mitochondrial factors cytochrome c and apoptosis inducing factor (AIF) were found to be released into the cytosol as a major mechanism of apoptosis [19,20]. This was the beginning of a dramatically revised conception of mitochondria as a stress-responsive network integrally involved in a range of cell-wide responses. In addition to its now well-defined role in apoptosis, mitochondria are revealed to have critical mechanistic roles in cellular homeostasis pathways including unfolded protein response (UPR), autophagy, and inflammation. Mitochondrial dysfunction activates UPR and remodels chromatin in the nucleus, altering histone methylation to direct stress response gene expression [21], while loss of Δψ_m_ causes PTEN-induced kinase-1 (PINK1) recruitment for Parkin-mediated autophagic degradation [22,23]. The simultaneous advance of our understanding of mitochondrial fission/fusion dynamics has demonstrated that the loss of mitochondrial interconnection often (though not always) precedes or accompanies cell signaling including apoptosis and autophagy, with fusion and fission factors often playing direct mechanistic roles in these cascades. Mitochondrial fission emerged as a key mechanistic step in cytochrome c release and apoptotic induction [24] and is required for mitochondrial autophagy [25,26], while increased mitochondrial fusion protects against apoptosis [27] and prevents targeting of mitochondria for autophagic degradation [28]. Conversely, loss of mitochondrial fusion activates inflammatory signaling [29,30].

The revelation of mitochondria as a pleiomorphic, responsive network prompted a very exciting era of discovery as the factors behind mitochondrial dynamics were identified and characterized. Currently, the OMA1 metalloprotease and dynamin-related protein-1 (DRP1) are emerging as highly responsive proteins that mediate stress-sensitive regulation of mitochondrial fusion and fission, respectively. Dysregulation of mitochondrial dynamics is emerging as a powerful underlying component of the pathology of prevalent human diseases, as demonstrated by patient-based and experimental models of cardiomyopathy [31,32,33] and neurodegeneration [34,35], among others. Increased understanding of mitochondrial dynamics and their response to cellular stress will drive forward our ability to prevent and interdict cellular pathology among some of the most pervasive diseases confronting society.

## 4. Mitochondrial Fusion Homeostasis is Regulated by OMA1

Mitochondrial fusion requires distinct factors responsible for the outer and inner membranes. Mitofusins 1 and 2 are required for outer membrane fusion [36], with fusion of the inner membrane accomplished by optic atrophy-1 (OPA1), which was identified as a causative gene in dominant optic atrophy [37], and subsequently shown to play a vital role in mitochondrial dynamics. While outer and inner membrane fusion are separate events, OPA1-mediated inner membrane fusion requires that mitofusin 1-mediated outer membrane fusion occurs first, followed by fusion of the inner membrane [38]. Upon the serial fusion of the outer and inner membranes, the fused mitochondria become a continuous organelle, allowing free diffusion of both matrix and membrane-bound proteins throughout the previously separate organelles. Critically, fusion of the inner membrane requires an intact Δψ_m_ [12]. This Δψ_m_-dependent fusion is modulated by proteolytic cleavage of OPA1.

**Figure 2:**
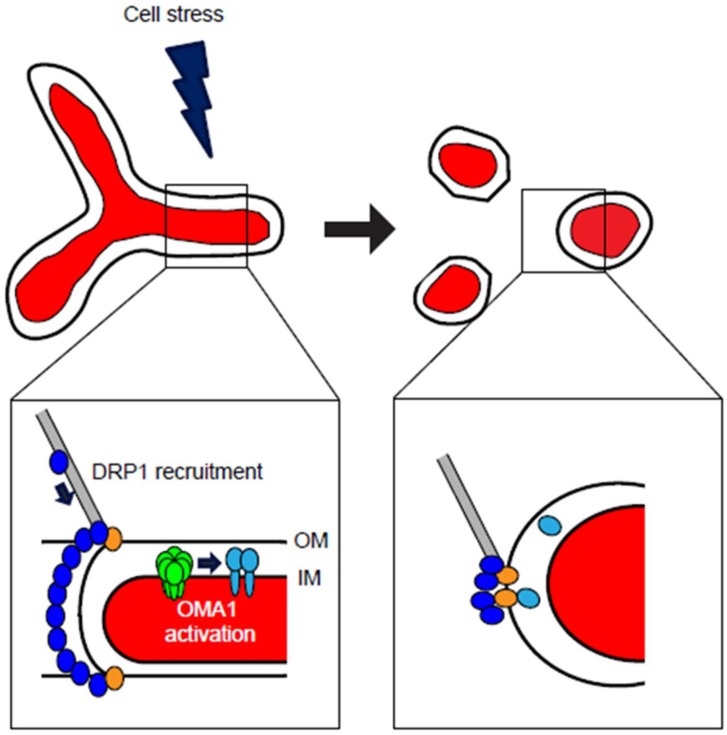
Cellular stresses induce mitochondrial fragmentation through DRP1- and OMA1-dependent mechanisms. At steady-state conditions, mitochondria maintain a balance of fusion and fission, with ability of organellar interconnection (top left). Cell stress, including cytokines, oxidants, bioenergetic deficits, or other stimuli, can activate either DRP1-mediated mitochondrial fission, OMA1-mediated loss of mitochondrial fusion, or both. Recruitment of DRP1 (blue) to the organelle is accomplished through cytoskeletal trafficking, where DRP1 is bound by mitochondrial receptors including MFF, Fis1, MiD49, and MiD51 (gold) at the outer membrane (OM) (detail image, lower left). At the inner membrane (IM), stresses that decrease Δψ_m_ may activate homo-oligomeric OMA1 (green), which responds by cleaving OPA1 (light blue) from its fusion-active L-OPA1 isoforms to the fusion-inactive S-OPA1. Upon activation of these responses, mitochondrial form is remodeled to a fragmented morphology (top right), in which neither OM nor IM are interconnected. Following DRP1 multimeric constriction and division of the organelle, DRP1 remains localized at punctate foci with its mitochondrial receptor proteins, while the cleaved S-OPA1 is released from the inner membrane into the intermembrane space, with some S-OPA1 colocalizing at DRP1 fission sites (detail image, lower right).

As demonstrated by its role as the underlying mechanism of dominant optic atrophy, OPA1 is a crucial mediator of bioenergetic-linked mitochondrial dynamics. As such, OPA1 has a complex set of regulatory mechanisms at both the transcriptional and post-translational levels, with alternative splicing variants and proteolytic cleavage playing critical roles in the regulation of OPA1-mediated mitochondrial fusion. The expression of human *OPA1* is highly complex, as the *OPA1* gene contains 31 exons that undergo variable splicing to produce 8 mRNAs of varying length, determined by the inclusion or exclusion of the 4, 4b, and 5b exons [39]. This results in distinct expression patterns showing the highest levels of *OPA1* expression in metabolically-demanding tissues such as retina, brain, and heart [40,41,42]. This intricate set of eight *OPA1* mRNA isoforms provide a set of distinct functionalities at the protein level in maintaining mitochondrial dynamics. These mRNA splice variants are translated and imported into mitochondria, where the mitochondrial targeting signal is cleaved and the protein inserted into the inner membrane [43] appearing as the two long isoforms of OPA1 (L-OPA1) that are responsible for mediating inner membrane fusion. As such, OPA1 is tightly regulated by the proteolytic cleavage of L-OPA1 at the S1 and S2 cleavage sites, producing a mix of long L-OPA1 and short S-OPA1 comprising five distinct protein isoforms, ranging from ~85–100 kDa and appearing as discrete bands via Western blot. Constitutive OPA1 proteolysis occurs at the S2 cleavage site, mediated by the intermembrane space AAA (*i*-AAA) protease YME1L and resulting in steady-state production of the S-OPA1 isoforms [27,44]. OPA1’s S1 cleavage site, however, is inducible: the loss of L-OPA1 in response to mitochondrial depolarization indicated that an unidentified protease was activated in response to loss of Δψ_m_. The groups of van der Bliek [45] and Langer [46] concurrently identified OMA1 as the major mediator of Δψ_m_-sensitive OPA1 cleavage. Importantly, this balance of long, fusion-active and short, fusion-inactive OPA1 is Δψ_m_-sensitive: loss of Δψ_m_ causes cleavage of L-OPA1 and accumulation of S-OPA1, resulting in mitochondrial fragmentation [47,48]: the three short forms S-OPA1 forms are released into the intermembrane space, thus rendering them fusion-inactive [47]. Moreover, while S-OPA1 isoforms are fusion-inactive, they have been suggested to play important mechanistic roles in activating fission, as exogenous S-OPA1 increase fission and partially localizes to mitochondrial fission sites in mouse embryonic fibroblasts (MEFs) [27] (Figure 2) and appear to regulate mitochondrial bioenergetics function and cristae structure [49]. Thus, both long and short OPA1 isoforms have important, yet distinct, functional roles in mitochondrial dynamics and homeostasis. The balance of long, fusion-active and short, fusion-inactive OPA1 is carefully regulated by proteolytic cleavage via the inner membrane proteases YME1L and OMA1.

The balancing of long and short OPA1 isoforms is a critical mechanism of mitochondrial dynamic homeostasis, controlled by splicing, Dym, and constitutive cleavage by YME1L [44]. Full-length L-OPA1 isoforms, inserted in the membrane, maintain a fused inner membrane either through homotypic OPA1:OPA1 interactions or through interaction with cardiolipin [50]. While both long L-OPA1 and short S-OPA1 are required for effective mitochondrial fusion [44], once the long L-OPA1 isoforms are cleaved to short S-OPA1, mitochondria can no longer maintain interconnection. Previously identified in yeast as a mitochondrial metalloprotease [51], mammalian OMA1 is activated in response to loss of Δψ_m_, cleaving L-OPA1 and causing fragmentation of the mitochondrial network [45,46]. Intriguingly, OMA1 undergoes self-cleavage [52] and degradation [53] following activation through loss of Δψ_m_. Thus, Δψ_m_-dependent mitochondrial fusion involves a complex set of interactions among proteins located at the mitochondrial inner membrane. Upon loss of Δψ_m_, OMA1 is activated, likely through a short N-terminal Δψ_m_-sensing domain [54], upon which it cleaves L-OPA1, resulting in loss of mitochondrial fusion (Figure 2). This is closely followed by OMA1’s degradation [53]. A variety of stressors have been shown to activate OMA1-dependent L-OPA1 cleavage in mouse embryonic fibroblasts, including CCCP, valinomycin, oligomycin, heat, and hydrogen peroxide (H_2_O_2_) [54], as well as in human HEK293T, SHSY5Y and HeLa lines, as well as murine N2a cell settings [53,55]. Intriguingly, inflammatory insults such as tumor necrosis factor-alpha (TNF-α) and lipopolysaccharide (LPS) induce overall degradation of OPA1, rather than L-OPA1 cleavage, in mouse and rat models; this OPA1 degradation was nevertheless abrogated by OMA1 knockdown [31]. L-OPA1 is also destabilized by loss of associated factors including AFG3L2 [56] and DRP1 [57,58].

## 5. DRP1 Regulates Stress-induced Mitochondrial Fission

Mitochondrial division, or fission, is mediated by DRP1, a highly conserved GTPase that is found throughout the cytoplasm of mammalian cells. DRP1 plays an analogous role to OMA1 in sensing cellular stimuli that alter dynamics, activating the mitochondrial fission apparatus, rather than the proteolytic disruption of fusion shown by OMA1, in response to external stimuli. Dynamins are inducible guanine triphosphatases (GTPases) that are well-characterized for their ability to constrict membranes to pinch off vesicles [59]; DRP1 carries out this function in dividing mitochondria by forming a multimeric collar around the organelle that constricts, effectively pinching the organelle in half [60].

First identified in yeast [61,62], mammalian DRP1 was localized to mitochondria and found to coalesce at mitochondrial division sites. When mitochondria are in fission/fusion balance, DRP1 is distributed throughout the cytoplasm, with some DRP1 localized to mitochondria at discrete punctate foci. Upon a shift to widespread mitochondrial fission, however, DRP1 is strongly recruited to the mitochondrial outer membrane, where DRP1 forms multimeric rings that catalyze the constriction and division of the organelle, similar to the canonical dynamins [60]. This organellar recruitment of cytosolic DRP1 is mediated by an array of DRP1-binding receptor factors at the mitochondrial outer membrane. Fis1 was identified as an outer membrane-localized mitochondrial receptor for DRP1 [63,64] followed by Mff [65]. More recently, MiD49 and MiD51 were identified as factors that recruit DRP1 independently of Fis1 and Mff [66,67]. In order to for recruitment to mitochondrial receptors, however, DRP1 must first get there. While actin had been proposed as a mechanism behind ER-mitochondrial contacts and fission [68], more recent studies reveal that DRP1 is transported to mitochondria through protein-protein interaction with actin, directly binding DRP1 and targeting it to the mitochondrial outer membrane as actin cycles onto and off of mitochondria [69]. This causes the accumulation and multimerization of DRP1 at discrete mitochondrial fission sites [70,71]. Following recruitment, DRP1 forms a multimeric ring around the division site and mechanically constricts to pinch the mitochondrial tubule in two (Figure 2): recent structural studies show that GTP hydrolysis induces allosteric constriction of DRP1, demonstrating the GTP-dependent mechanical force of DRP1 [72]. DRP1 performs the initial constriction of the organelle to ~100 nm, while dynamin-2 (dyn2), previously associated chiefly with endocytosis [73], performs the final ‘cut’ dividing the organelle in two. Thus, mitochondrial fission is driven by DRP1 and its coordinate interaction with multiple mitochondrial factors, acting both as receptors for DRP1 at the outer membrane and for sequential constriction/division of the organelle.

While the stress response of OMA1 centers on its ability to sense perturbations in Δψ_m_ as an activating mechanism for L-OPA1 cleavage and mitochondrial fragmentation, DRP1-mediated stress response has a more varied range of activation mechanisms due to the multiple mitochondrial receptors and cytoskeletal elements involved in DRP1 mitochondrial fission. As such, DRP1-mediated fission is activated not only in response to loss of Δψ_m_, but also in response to other bioenergetic deficits and cell signaling pathways. For example, while uncouplers such as CCCP activate mitochondrial fragmentation via OMA1-mediated cleavage of OPA1, rotenone and antimycin A, both pharmacological inhibitors of oxidative phosphorylation, do not cause OPA1 cleavage in human U2OS cells, but instead activate mitochondrial DRP1 recruitment and fission. This DRP1-mediated fission is 5’ AMP-activated kinase (AMPK)-dependent, as the loss of bioenergetic function activates AMPK to phosphorylate MFF [74]. The roles of MFF, Fis1, MiD49, and MiD51 as mitochondrially-located DRP1 receptors provides a ready mechanism for distinct roles for each in promoting DRP1-mediated mitochondrial fission. Indeed, while both MFF and MiD51 associate with DRP1 in close proximity in MEFs, each appears to have specific roles in activating DRP1 recruitment to the organelle [75].

In addition to the activation roles of the multiple mitochondrial receptors for DRP1, DRP1 itself is regulated by multiple post-translational modifications, providing critical signaling inputs to activate DRP1’s recruitment for mitochondrial division. The phosphorylation of DRP1 at Ser 656 was a key mechanistic insight, demonstrating a post-translational modification that directly inhibits DRP1-mediated mitochondrial fragmentation in rat PC12 cells [76], with the analogous human DRP1 Ser 637 showing the same phosphorylation-dependent inhibition [77]. Phosphorylation of DRP1 at this site inactivates DRP1’s GTPase activity and mitochondrial recruitment, causing mitochondrial elongation. Conversely, phosphorylation of rat DRP1 at Ser 585 promotes mitochondrial fission as part of mitosis [78]. These findings indicated that the phosphorylation profile of DRP1 provides a delicate mechanism for regulation of mitochondrial fission. Consistent with this, kinase/phosphatase activity by a wide range factors has been shown to modulate DRP1 phosphorylation and mitochondrial fission, with calcineurin [79], protein kinase A [77], PP2/Bβ2 [80], Erk2 [81] all modulating DRP1 phosphorylation at various residues. In addition, SUMOylation of DRP1 by SENP5 activates mitochondrial fission [82,83] and facilitates cytochrome c release for apoptosis [84]. These post-translational modifications provide crucial activation of DRP1 stress response mechanisms. In addition, the interaction of DRP1 with the cytoskeleton provides yet another level for potential regulation of stress response: HeLa cells respond to high salt and oxidative stress conditions via hyperacetylation of microtubules and DRP1 phosphorylation to drive mitochondrial fragmentation [85].

## 6. Open Questions

The molecular machinery of both DRP1 and OMA1 is an emerging field. While the cleavage of OPA1 by OMA1 [45,46,52] and the actin-mediated mitochondrial recruitment [70,71] and active organellar constriction of DRP1 [72] are critical mechanistic insights, a better understanding of the activation of both processes by oxidants and cellular stressors is essential to develop a more comprehensive picture of how OMA1 and DRP1 maintain mitochondrial dynamic homeostasis. As mitochondrial dynamics are increasingly emerging as upstream determinants of stress responses including inflammation, autophagy, and apoptosis, understanding the sensitive regulation of mitochondrial fusion/fission homeostasis will be critical to interdicting these responses in a wide range of tissue-specific pathologies.

## 7. Activation and Domain Mapping of OMA1 and DRP1

The phosphorylation status of DRP1 at its key residues provides a crucial mechanism for control of mitochondrial division. The activation and inhibition of DRP1 through phosphorylation at conserved serine residues, followed by actin-mediated recruitment to the mitochondrial binding partners at the outer membrane, shows that mitochondrial fission is subject to regulation at multiple steps before the organelle is divided. Future work may involve a more comprehensive exploration of the signaling pathways that specifically mediate DRP1 phosphorylation and mitochondrial recruitment, parsing the specificity or globality of serine phosphorylation as a control mechanism. It will also be critically important to determine how oxidative insults and cytokines impact DRP1 phosphorylation, and through which pathways, as this activation of mitochondrial fission is a likely contributor to activation of inflammation and apoptosis in a variety of pathological contexts. Increased fission may also help protect the cell from cellular stress: RhoA activates DRP1 through phosphorylation at Ser 616, protecting rat cardiomyocytes from cell death [86]. These findings demonstrate that a complex web of signaling events regulate DRP1-mediated mitochondrial fission, and that this may be deleterious or beneficial, depending on context. In a more cell-wide view, it will be intriguing to determine the point at which DRP1 causes fission of the entire mitochondrial network: having identified the sequence of interactions necessary for individual mitochondria to divide as part of fission/fusion homeostasis, at what point does DRP1 tip the balance of mitochondrial structure to a completely fragmented state? The interactions and signaling inputs identified for DRP1 provide a mechanistic framework from which to approach these key integrative questions.

Future work is needed to more comprehensively understand how OMA1 is activated by distinct stress stimuli. While the M48 metalloprotease domain of OMA1 is responsible for proteolytic cleavage, a short N-terminal (a.a. 144–163) transmembrane potential sensor domain, predicted to lie on the matrix side of the inner membrane, is required for CCCP-induced OPA1 cleavage [54]. The regulation and activation of the M48 domain remains to be elucidated. How does the 144–163 stress-sensor domain activate cleavage by the M48 domain? The CCCP-induced change in voltage or chemical environment at the stress-sensor domain may cause a conformational shift in OMA1, allowing the M48 domain access to L-OPA1, permitting cleavage to S-OPA1. It is likely that the N-terminal region of OMA1 provides differential regulation of OMA1 activity in response to a variety of stimuli that cause OPA1 cleavage, such as Δψ_m_ uncouplers CCCP and FCCP, ATP depletion, oxidative stressors, cytokines, and others. Moreover, the data support differential activation mechanisms of OPA1 degradation: while uncoupling stresses (i.e., those that diminish Δψ_m_) cause L-OPA1 cleavage to S-OPA1, cytokine-mediated stresses may actually cause total degradation of both L- and S-OPA1 isoforms, as seen in cardiomyoblasts treated with TNF-α [31]. L-OPA1 isoforms are increasingly shown to correlate with anti-apoptotic cell survival, deterring the onset of neurodegeneration [34] and aging [30]. As such, the activation of OMA1 proteolytic cleavage is a critical tipping point that needs better mechanistic knowledge. Improved structural understanding of OMA1 and its key domains, both at rest and upon activation, will fill a major gap in knowledge regarding this critical stress sensor.

## 8. Transcriptional Regulation

Given the emerging importance of OMA1 and DRP1 to mitochondrial homeostasis and downstream stress signaling response, improved understanding of the regulation of their expression is needed. The levels of DRP1 and OPA1 change in response to a range of stimuli; however, a more comprehensive picture of regulation of key fission/fusion factors in response to signaling events is needed, both in terms of their transcriptional regulation and the impact those changes have on mitochondrial dynamics.

Fiorenza et al. showed that *DRP1* mRNA levels are increased in skeletal muscle in response to exercise, coordinately upregulated with other mitochondrial control genes including nuclear respiratory factor-2 (*NRF2*), peroxisome proliferator-activated receptor gamma coactivator-1 alpha (*PGC-1α*), and transcription factor A, mitochondrial (*TFAM*) [87]. Consistent with this, Ding et al. found that PGC-1α binds directly to the DRP1 promoter to activate transcription [88]. These studies indicate that transcription of DRP1 is, at least in some contexts, regulated together with other targets of the mitochondrial biogenesis regulatory pathways. This broad regulation of mitochondrial content is likely partly overlapping, but partly distinct, from stress-induced regulation. For example, several groups have demonstrated that DRP1 transcription is differentially altered in response to various stimuli: DRP1 levels are increased in response to oxidative stress [89,90], manganese [91], and bisphenol A [92], suggesting that DRP1 transcriptional regulation is sensitive to both stress response and mitochondrial biogenesis programs. 

First identified as a nuclear gene on Chromosome 3 [37], OPA1 transcription appears to be altered in response to both PGC-1α mitochondrial biogenesis and NF-KB stress cues. Stresses including manganese [91] cause decreased OPA1 levels. Insights into specific regulation of OPA1 have focused on dissecting the promoter region immediately upstream. NF-KB signaling allows NEMO to bind to the OPA1 promoter region, mediating Parkin-dependent upregulation of OPA1 [93]. Similarly, Stat3 and RelA bind to respective binding sites on the OPA1 promoter, upregulating expression independently of PGC-1α-driven mitochondrial biogenesis [94]. OPA1 expression is decreased in mice lacking PGC-1α [95], consistent with a role for PGC-1α-directed regulation of OPA1 expression [96]. OMA1 transcription is less well explored; however, the murine OMA1 contains an estrogen-response element in its promoter region [97]. These findings collectively indicate that the levels of critical fusion and fission factors are dramatically altered by specific mechanisms in response to cellular signaling.

It is important to bear in mind that mitochondrial dynamics exist as a delicate homeostatic balance, in which either excessive fusion *or* fission is detrimental to overall cellular metabolism and viability. While the collapse of mitochondrial interconnection represented in Figure 2 is the most commonly-observed disruption of mitochondrial fission/fusion homeostasis, pushing the balance too far in either direction is likely to be detrimental to the cell. Cells that have decreased expression of DRP1 show mitochondrial dysfunction [98,99], as do cells with loss or knockdown of OPA1 or OMA1 [100]. Despite the critical balance of mitochondrial dynamics, however, sensitive modulation of OMA1 and OPA1 levels may have provocative impact in protecting cells against various stressors. Exciting recent findings show that decreasing expression of OMA1 in murine models of neurodegeneration stabilizes the mitochondrial network against fragmentation and prevents loss of mtDNA, delaying neuronal death and preserving lifespan [34]. The specific transcriptional regulation of OMA1, OPA1, and DRP1, and the resulting impact of the change in expression levels on mitochondrial dynamic balance, presents an exciting challenge to the field.

## 9. Conclusions

The sensitive homeostatic balance of mitochondrial fission/fusion dynamics, determined by stress-sensitive action of OMA1 and DRP1, is emerging as a critical upstream determinant of cellular stress responses through direct mechanistic connections, particularly in energetically-demanding cell types, such as skeletal muscle [29]. Improved understanding of the activation and regulation of mitochondrial fission/fusion homeostasis, particularly the OMA1 and DRP1 factors, will provide vital insights into this early indicator of cellular stress as part of the underlying pathology of pervasive disorders including neurodegeneration, diabetes, cardiovascular disease, and aging. Translationally-directed modulation of mitochondrial dynamics mediated by OMA1 and DRP1 presents an exciting opportunity to prevent or rescue cellular pathology across this range of prevalent human diseases.
